# O-GlcNAcylation in Chronic Lymphocytic Leukemia and Other Blood Cancers

**DOI:** 10.3389/fimmu.2021.772304

**Published:** 2021-11-18

**Authors:** David E. Spaner

**Affiliations:** ^1^ Biology Platform, Sunnybrook Research Institute, Toronto, ON, Canada; ^2^ Department of Immunology, University of Toronto, Toronto, ON, Canada; ^3^ Department of Medical Biophysics, University of Toronto, Toronto, ON, Canada; ^4^ Department of Medical Oncology, Sunnybrook Odette Cancer Center, Toronto, ON, Canada; ^5^ Department of Medicine, University of Toronto, Toronto, ON, Canada

**Keywords:** O-GlcNAc transferase (OGT), O-GlcNAcase (OGase), cancer, metabolism, chronic lymphocytic leukemia, signal transduction, cytokines, O-linked β-D-N-acetylglucosamine (O-GlcNAc)

## Abstract

In the past decade, aberrant O-GlcNAcylation has emerged as a new hallmark of cancer. O-GlcNAcylation is a post-translational modification that results when the amino-sugar β-D-N-acetylglucosamine (GlcNAc) is made in the hexosamine biosynthesis pathway (HBP) and covalently attached to serine and threonine residues in intracellular proteins by the glycosyltransferase O-GlcNAc transferase (OGT). O-GlcNAc moieties reflect the metabolic state of a cell and are removed by O-GlcNAcase (OGA). O-GlcNAcylation affects signaling pathways and protein expression by cross-talk with kinases and proteasomes and changes gene expression by altering protein interactions, localization, and complex formation. The HBP and O-GlcNAcylation are also recognized to mediate survival of cells in harsh conditions. Consequently, O-GlcNAcylation can affect many of the cellular processes that are relevant for cancer and is generally thought to promote tumor growth, disease progression, and immune escape. However, recent studies suggest a more nuanced view with O-GlcNAcylation acting as a tumor promoter or suppressor depending on the stage of disease or the genetic abnormalities, proliferative status, and state of the p53 axis in the cancer cell. Clinically relevant HBP and OGA inhibitors are already available and OGT inhibitors are in development to modulate O-GlcNAcylation as a potentially novel cancer treatment. Here recent studies that implicate O-GlcNAcylation in oncogenic properties of blood cancers are reviewed, focusing on chronic lymphocytic leukemia and effects on signal transduction and stress resistance in the cancer microenvironment. Therapeutic strategies for targeting the HBP and O-GlcNAcylation are also discussed.

## Introduction

**Figure 1:**
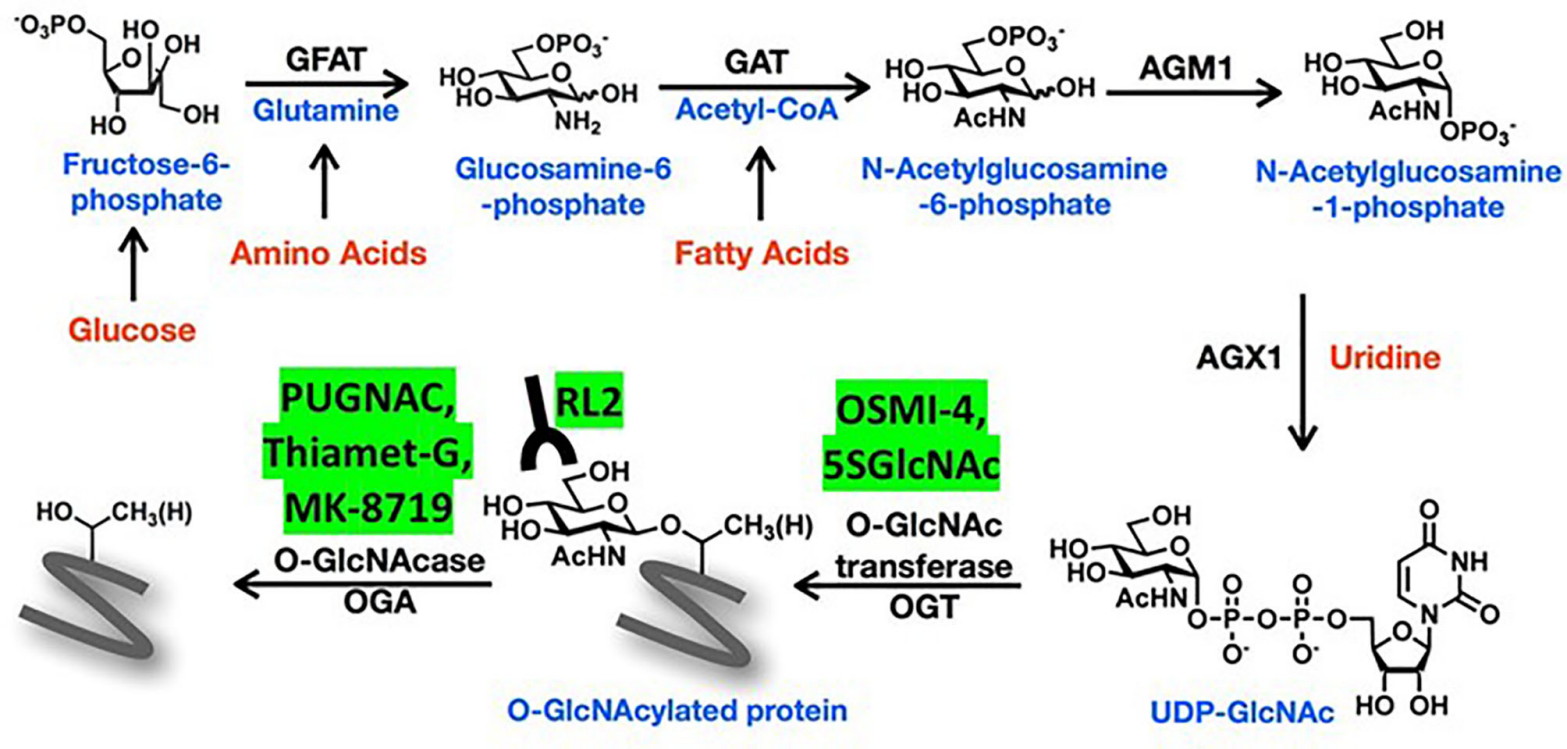
Schema of the hexosamine biosynthetic pathway (HBP). The amino sugar UDP-GlcNAc is generated in the HBP by combining fructose-6-phosphate, glutamine, nucleotides, and acetyl-CoA. More detailed description of the sequential enzyme reactions in the pathway is provided in the text. O-GlcNAc transferase (OGT) uses UDP-GlcNAc to transfer O-GlcNAc moieties onto target proteins that can be recognized by RL2 antibodies and removed by O-GlcNAse (OGA). OGT and OGA inhibitors mentioned in the text are shown in the yellow boxes.

Upon entry into a cell, glucose is phosphorylated to fructose-6-phosphate before continuing down the glycolysis pathway. About 2-5% of fructose-6-phosphate is normally diverted into the hexosamine biosynthetic pathway (HBP) (**Figure 1**), a minor metabolic pathway increasingly recognized to have an important role in cancer biology (1). The rate-limiting enzyme of the pathway is glutamine fructose-6-phosphate amidotransferase (GFAT), which has two isoforms (GFAT1 and GFAT2 encoded by *GFPT1* and *GFPT2*, respectively) and generates glucosamine (GlcN)-6-phosphate from fructose-6-phosphate and glutamine. Acetyl-CoA is added by GlcN-6-phosphate acetyl transferase (GNAT) to make N-acetyl glucosamine (GlcNAc)-6-phosphate, which is rearranged to GlcNAc-1-phosphate by GlcNAc-phosphoglucomutase (AGM). GlcNAc-1-phosphate Pryophosphorylase (AGX) adds uridine-diphosphate (UDP) to ultimately form the nucleotide-sugar uridine diphosphate N-acetylglucosamine (UDP-GlcNAc). UDP-GlcNAc is involved in glycosylation of cell-surface lipids and proteins (2) but also employed by O-linked GlcNAc transferase (OGT) to O-GlcNAcylate serine and threonine residues on intracellular proteins. These modifications can be removed by the deglycosylating enzyme, O-GlcNAcase (OGA) encoded by *OGA*, formerly known as *MGEA5* (2, 3) (**Figure 1**).

The biological activity of thousands of different proteins can be altered by O-GlcNAcylation (4). Serine and threonine targets of OGT may be phosphorylation sites that are blocked by O-GlcNAc residues to disturb signaling pathways (1). Changes in protein structure by O-GlcNAcylation can also promote phosphorylation and alter cellular localization as well as protein-protein interactions and formation of supramolecular complexes including transcriptional regulators that produce changes in gene and protein expression. Cross-talk between O-GlcNAcylation and ubiquitination can modulate protein expression by either preventing or promoting proteasomal degradation (5).

Antibodies like RL-2 and CTD110.6 (6) are used to detect O-GlcNAc on proteins since antibodies to O-GlcNAcylated forms of specific proteins are generally unavailable (7). Strategies to manipulate O-GlcNAcylated proteins include the use of different concentrations of glucose and glutamine (8) or GlcN and uridine in tissue culture media (9). Along with genetic modification, OGA can be blocked by PUGNAC or Thiamet-G (TMG), and OGT by small molecules like OSMI-4 (10) and metabolic inhibitors like 2-deoxy-2-N-hexanamide-5-thio-d-glucopyranoside (5SGlcNHex) (11, 12) (**Figure 1**). The glutamine analogue 6-diazo-5-oxo-L-norleucine (DON) is often used in the literature as a GFAT inhibitor but it has multiple substrates that may confuse biological interpretation of the data (13).

Aberrant O-GlcNAcylation occurs in a variety of human diseases including diabetes and dementia (1, 2). O-GlcNAcylation is induced by stressful conditions and thought to act as a cellular survival mechanism (14, 15). Changes in O-GlcNAcylation from altered metabolism or stress can also dysregulate cell signaling networks (1, 14). Since cancer is the result of oncogenic events that cause replicative, proteotoxic, nutrient, and/or oxidative stresses (16, 17) and is driven by dysregulated signaling pathways (18), it is not surprising that high levels of O-GlcNAcylated proteins also characterize and are involved in the basic hallmarks of cancer (19, 20).

O-GlcNAcylation has been studied mainly in solid tumors where it is linked to enhanced glycolysis and aggressive clinical behavior (21). Its effects on transcription factors in cancer have also been reviewed recently (22–24). The purpose of this review is to discuss recent information regarding O-GlcNAcylation in signaling and survival (25) of cancer cells in the context of chronic lymphocytic leukemia, the first blood cancer noted to exhibit aberrant O-GlcNAcylation (2). Studies about O-GlcNAcylation in other blood cancers are also reviewed.

## Chronic Lymphocytic Leukemia

CLL is the most common adult leukemia with an incidence of 4.9/100,000 new cases per year in the US (26). It is a cancer of CD19^+^ B cells that co-express the T cell marker CD5. Males are twice as likely as females to develop CLL and at risk for more aggressive disease (27, 28).

Like any cancer, CLL passes through stages of initiation, promotion, and progression (29, 30). It is initiated by genetic lesions that transform hematopoietic stem cells (31), immature B cells with immunoglobulin heavy chain variable (*IGHV*) genes in the germline configuration, or more mature antigen-experienced B cells that have undergone somatic hypermutation. Patients with unmutated *IGHV* disease are considered to have a more aggressive form of CLL (32).

**Figure 2:**
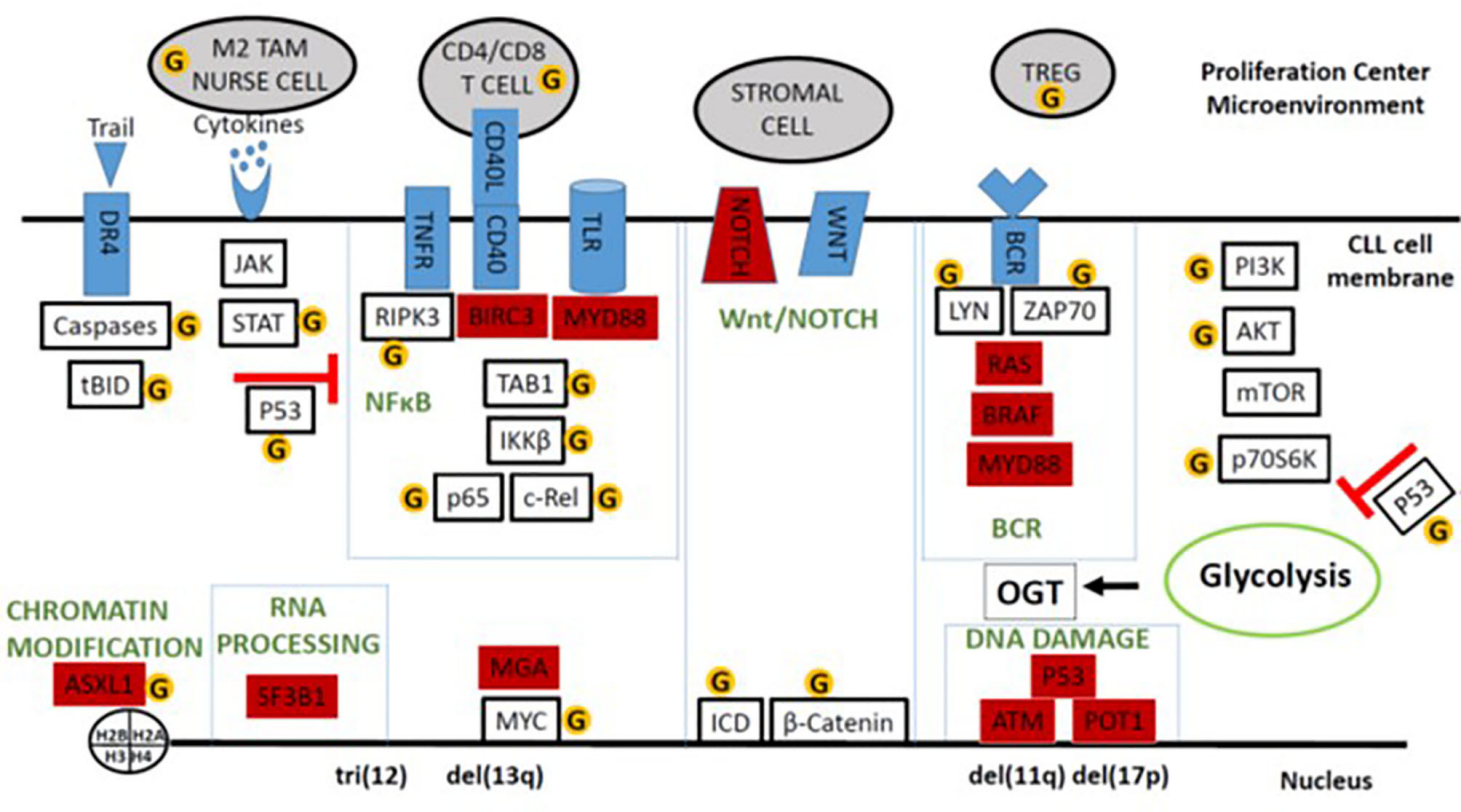
Schema of O-GlcNAcylated oncogenic processes in the CLL microenvironment. Major signaling and system modules that drive growth and progression of CLL cells are organized in the boxes along with important auxiliary pathways like cytokine- and AKT-signaling. Genes frequently mutated in CLL are colored in red. Proteins and cells discussed in the text that can be affected by O-GlcNAcylation are indicated by the yellow circled G’s. The figure emphasizes that O-GlcNAcylation increases the tumor-suppressing activity of wild-type p53 but enhances many of the signaling processes that support tumor growth in the presence of mutant p53 and an impaired p53 axis.

Promoters cause transformed cells to proliferate until they reach sufficient numbers to become clinically evident (33). Hypercholesterolemia may promote tumor growth by altering membrane lipid content and affecting signaling modules in CLL cells (34–36). Signals that drive CLL cells to proliferate are delivered in proliferation centers (PCs) found in lymphoid organs (37) (**Figure 2**). Proliferative signals are transmitted through the B cell receptor (BCR), Toll-like receptors (TLRs), tumor necrosis factor (TNF) receptors like TNFR1 and TNFR2 (38), non-canonical TNF receptors like CD40, cytokines like type 1 and type 2 interferon (IFN), IL2, IL4, IL15, and IL21 (39–41), and NOTCH family members, particularly NOTCH 1 and 2 (42). Consequently, important signaling pathways in CLL include the NFκB, MEK/ERK, PI3K/AKT/mTOR, janus-kinase (JAK), and NOTCH pathways that can all be affected by O-GlcNAcylation (**Figure 2**).

Resting CLL cells traffic from blood to PCs in response to chemokines and their receptors such as CXCR4 (43). CXCR4^hi^CD5^lo^ cells in blood are about to re-enter PCs while CXCR4^lo^CD5^hi^ CLL cells are recent emigrants that retain some of the transcriptional program in that microenvironment (44). T cells, macrophages, stroma, as well as other leukemia cells produce the cytokines, TLR-ligands, and antigens in PCs that stimulate CLL cells (37). Macrophages in the CLL microenvironment are called “nurse” cells with properties of anti-inflammatory M2 macrophages (45, 46). They support leukemic growth and survival by making factors like the TNF-family member BAFF (45) and through direct protein-protein interactions (47). Nurse cells also impair effective anti-tumor immunity and prevent clearance of CLL cells by secreting immunosuppressive factors such as IL10 (46). Interactions of CLL cells and myeloid cells in the microenvironment cause expansion of regulatory T cells (Tregs) (48, 49) that contribute to immunosuppression and facilitate leukemic growth (**Figure 2**).

## Heterogeneity of CLL

Despite a common CD5^+^CD19^+^ phenotype, CLL is marked by a heterogeneous clinical course ranging from a benign disease to one that can be fatal within a few years of diagnosis (32). Cytogenetic abnormalities in CLL cells distinguish 5 major sub-types. Trisomy 12 is found in 10-20% of patients while 40-50%, 10-12%, and 10-15% have deletions at 13q14, 11q22-q23, and 17p13, respectively, and 20-30% of patients display no apparent abnormalities with standard probes (32). Prior to the advent of novel therapies such as kinase and Bcl-2 inhibitors (49, 50), disease severity was associated with these abnormalities with del(17p) > del(11q) > trisomy 12 > normal cytogenetic analysis > isolated del(13q). Other factors associated with aggressive disease include an unmutated *IGHV* gene suggesting origin in an earlier stage of B cell development, advanced clinical stage, short lymphocyte doubling time *in vivo*, and high ZAP70 and β2-microglobulin levels (32). Genomic studies have identified over 40 driver mutations in genes such as *NOTCH1, SF3B1, ATM, p53*, *BIRC3, POT1, BRAF, MGA* and *MYD88* that emphasize the diversity of CLL and affect prognosis (**Figure 2**) (16).

Dysregulated signaling is central to the biology of CLL. Enhanced responses of leukemia cells to BCR- or TLR-agonists *in vitro* are associated with aggressive clinical behavior and thought to reflect the events in PCs *in vivo* (51, 52). Cytokine signaling is also corrupted in clinically aggressive CLL cells that harbor mutations of *ATM* or *TP53*. Type 1 IFN inhibits growth of indolent CLL cells and activates the canonical signaling pathway, characterized by prolonged phosphorylation of STAT1 and brief phosphorylation of STAT3. In aggressive CLL cells, IFN causes prolonged STAT3 phosphorylation associated with immunosuppressive factor production and tumor growth *in vitro* (53).

Many driver mutations associated with more aggressive disease do not activate CLL cells directly but serve to amplify responses to proliferative signals in the CLL microenvironment (16) (**Figure 2**). *NOTCH* mutations characteristically prolong the life of the intracellular domain (ICD) that mediates transcription, producing enhanced NOTCH-signaling responses in more aggressive cells (54). Inactivating mutations of *BIRC3* produce exaggerated non-canonical NFκB responses (55). Activating mutations in members of the RAS-BRAF-MAPK-ERK pathway lead to enhanced signaling through the BCR and other growth factor receptors (56). *SF3B1* mutations promote mis-splicing of *MAP3K7* (TAK1), resulting in hyperactivation of NFκB by dysregulating TNFα and TLR-signaling (57). Activating *MYD88* mutations enhance signaling through TLRs but are often associated with more indolent disease (58) (**Figure 2**).

Aberrant activity of AKT and c-MYC are associated with more aggressive forms of CLL, including transformation into a drug-resistant large cell lymphoma called Richter’s transformation (RT) (59). Leukemia cells of patients with high-risk disease and RT express high amounts of activated phosphorylated AKT and a phenotype resembling RT resulted from constitutive activation of AKT in the Eµ-TCL1 CLL mouse model (59). The MYC repressor MGA is recurrently mutated in aggressive forms of CLL (60) and c-MYC activation is a feature of RT (61).

The state of the p53 axis helps clarify the classification of the different molecular sub-types into aggressive or indolent forms (62). P53 is the major tumor-suppressor in most cancers, controlling a myriad of pathways that repair DNA, inhibit glycolysis and proliferation, and promote cell death (63). CLL cells with deletions of chromosome 17 encoding *TP53* are associated with an aggressive disease that is resistant to cytotoxic chemotherapy (32). Other subtypes exhibit a similar disease phenotype where the underlying molecular lesions impair the p53 axis independent of mutational damage to *TP53*. For example, MDM2 negatively regulates p53 by marking it for rapid clearance by the proteasome (62). *MDM2* is located on chromosome 12 so that CLL cells with trisomy 12 have higher MDM2 levels causing decreased p53 levels and activity. ATM phosphorylates p53 through CHK2, preventing it from binding MDM2 and being degraded. CLL cells with deficient ATM activity would then have diminished levels and activity of p53 (63). POT1 is a component of the Shelterin complex that protects telomeres by activating p53. Inactivating POT1 mutations diminish the p53 axis and allow chromosomal instability of leukemia cells (64). In contrast, the p53 axis is generally intact in CLL cells with del(13q) and a more indolent course that is responsive to cytotoxic chemotherapy (53, 65). These observations suggest the disparate clinical behavior of CLL subtypes may reflect the underlying status of the p53 pathway (**Figure 2**).

## O-GlcNAcylation IN CLL

Aberrant O-GlcNAcylation may play a role in CLL at a number of stages in disease progression. The sex-based incidence and severity of CLL could in part relate to the location of the *OGT* gene on the X-chromosome (28, 66). Dietary habits that produce hypercholesterolemia may be linked to the development of CLL in part by increasing O-GlcNAcylation in contributory cell types (67).

Like other cancers, CLL cells are characterized by high levels of O-GlcNAcylated proteins (2). Interestingly, miR-15a on chromosome 13 targets *OGT* (68) and is deleted in a majority of CLL cells (69). CLL cells express only GFAT1 in contrast to circulating peripheral blood mononuclear cells (PBMCs) that express both isoforms. Total levels of O-GlcNAcylated proteins are significantly higher in CLL cells than PBMCs but the O-GlcNAcome is quite variable, as indicated by the number and density of bands on an immunoblot developed with RL2 antibodies (2). All CLL cells have high levels of O-GlcNAcylated proteins compared to normal lymphocytes but leukemia cells with a lower RL2 index, based arbitrarily on the intensity of all RL2-staining bands on a gel normalized to β-actin, were associated with more aggressive disease indicated by greater hematopoietic impairment, high doubling times, and genetic lesions such as del(11q)and del(17p) (2). This observation is somewhat paradoxical as higher O-GlcNAc levels are often associated with more aggressive clinical behavior in solid tumors (70). However, similar to these findings in CLL, OGT and O-GlcNAcylated protein levels were significantly lower in ovarian cancers resistant to chemotherapy compared to chemosensitive cancers (71).

**Figure 3:**
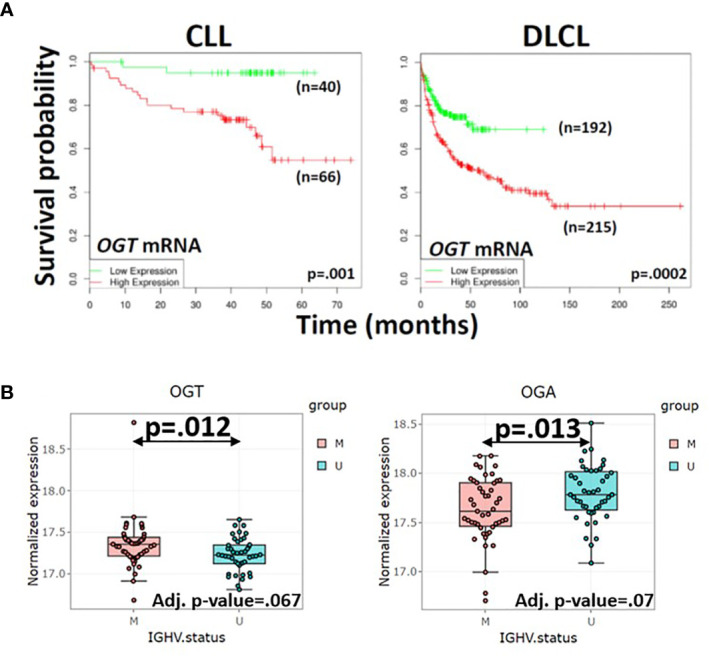
Correlation of *OGT* mRNA and protein levels in primary blood cancer cells with clinical course. **(A)** Survival of patients with high (n=66) or low (n=40) expression of *OGT* in CLL cells (left panel) and survival of diffuse large cell lymphoma patients with rituximab, cyclophosphamide, vincristine, adriamycin, and prednisone (R-CHOP) with high (n=215) or low (n=192) *OGT* expression in lymph node biopsies (right panel) were respectively compared by *in silico* analysis of the Herold (72) and Lenz (73) databases using the DRUGSURV bioinformatics analysis tool (http://www.bioprofiling.de/GEO/DRUGSURV). P-values for differences between the curves are 0.001 (left) and 0.0002 (right). **(B)** Expression of OGT (left) and OGA (right) proteins in 44 patients with aggressive CLL cells more likely to have an impaired p53 axis as indicated by unmutated *IGHV* genes (U) and 47 patients with more indolent CLL cells and mutated *IGHV* genes (M) were obtained with the R Shiny app (http://mozi.embl.de/public/proteomExplorer) provided for exploration of the CLL proteome (74). P-values and adjusted P-values are shown in the graphs and suggest more aggressive CLL cells have a lower OGT/OGA ratio, possibly consistent with relatively lower global O-GlcNAcylated protein levels.

Reasons for the inverse correlation between total O-GlcNAcylated protein levels and clinical course in CLL are unclear. While high *OGT* mRNA expression is associated with an adverse outcome (**Figure 3A**, left panel), the ratio of OGT to OGA proteins is lower in CLL cells with unmutated *IGHV* genes (U) compared to cells with mutated genes (M) (**Figure 3B**). Relative changes in glycosylation and deglycosylation could produce lower O-GlcNAcylated protein levels in more aggressive CLL cells and higher levels in more indolent cells (**Figure 3B**). Differential O-GlcNAcylated protein expression may also reflect proteomic differences (74). Aggressive cells have different numbers and species of proteins (74) that are targets for O-GlcNAcylation compared to indolent cells (75–77). For example, higher levels of ZAP70, a signaling molecule that is O-GlcNAcylated in activated human T cells (4), are found in aggressive CLL cells (32, 51). Similarly, O-GlcNAcylated forms of OGT, p53, c-MYC, and AKT along with UDP-GlcNAc, OGT, and OGA exhibited inter-patient variability but sample sizes were too small to be able to correlate with clinical behavior (2).

The O-GlcNAcome in PCs has not been studied but is likely to differ from the blood compartment due to activating signals in that microenvironment. O-GlcNAcylated proteins, OGT, and OGA were found only in the IgD^-^ fraction of normal tonsils, suggesting they are associated with a state of activation of human B cells in microenvironments that resemble PCs (2). Cellular activity including evidence of glycolysis also increased significantly in circulating CLL cells from patients administered the JAK inhibitor ruxolitinib (78, 79). This finding suggests cytokine-signaling through JAKs may negatively regulate O-GlcNAcylated protein levels but the nature of the cytokines and mechanism are currently unclear.

## Effects of O-GlcNAcylation on Oncogenic Signaling Pathways in CLL

### P53

The state of the p53 axis helps classify CLL cells (62) and may explain how O-GlcNAcylation can be associated with both aggressive and indolent disease. O-GlcNAcylation at Ser 149 stabilizes wild-type p53 by blocking ubiquitin-dependent proteolysis, facilitating nuclear localization, and transcription of p53 target genes (80, 81). Increased O-GlcNAcylation may then activate an intact p53 pathway to prevent cancer progression, compatible with an indolent clinical course.

In contrast, mutant p53 proteins appear to be unaffected by O-GlcNAcylation (81). Loss of p53 function can lead to upregulated glycolysis and enhanced O-GlcNAcylation by mass action. Impairment of upstream pathways causing disruption of the p53 axis may also result in increased glucose uptake, glycolysis, and HBP activity. For example, mutational inactivation of *ATM* contributes to cancer progression through a metabolic mechanism rather than its conventional role in DNA repair (82, 83). In the absence of p53, O-GlcNAcylation of other proteins as a result of increased HBP and OGT activity may increase their ability to promote tumor growth, potentially explaining how O-GlcNAcylation can also be associated with more aggressive clinical behavior of CLL cells.

### AKT

Similar to p53, the PI3K/AKT pathway can also be affected in diametrically opposed ways by O-GlcNAcylation (21, 84). AKT is activated partially by PDK1-mediated phosphorylation at Thr308 and fully by mTORcomplex2 (mTORC2)-mediated phosphorylation at Ser473 (2). In a number of cancers, including of B cell origin (85), increased O-GlcNAcylation and OGT expression promote PI3K/AKT pathway activity and inhibition of OGT decreases AKT phosphorylation and downstream activity. Activators of the AKT pathway like DDX5 or TCL1 are stabilized by O-GlcNAcylation (86) or induced by O-GlcNAcylated transcription factors (87, 88).

In contrast, use of GlcN to increase O-GlcNAcylated protein levels decreased AKT activity in circulating CLL cells, evidenced by diminished size and lowered phosphorylation at Thr308 (2). Similar results are seen in other cell-types such as adipocytes (89). Why O-GlcNAcylation increases AKT-activity in some conditions and inhibits it in others is not clear but may reflect glucose uptake by the cells. Glucose transporters are decreased when O-GlcNAcylation has inhibitory effects on AKT but upregulated in situations where O-GlcNAcylation promotes AKT activity (84). The proliferative state of the cell may account for different behaviors of glucose transporters. Proliferating cells such as cancer cell models or CLL cells in PC microenvironments are highly glycolytic whereas adipocytes and circulating CLL cells are non-proliferative. The latter use mainly fatty oxidation as a metabolic strategy that is turned off when they are induced to proliferate (90).

### NFκB

The theme of differential control of tumorigenesis by O-GlcNAcylation depending on the state of the p53 axis and metabolic state of the leukemia cell appears to also apply to NFκB (37). Several key molecules in the BCR-, TLR- and NFκB signaling pathways that drive the growth of CLL cells are targets for O-GlcNAcylation including LYN, IKKβ, p65, c-REL, and TAB1 (91–95). In the absence of p53, increased O-GlcNAcylation promotes phosphorylation of p65 on Ser536, which is critical for nuclear translocation of NFκB and expression of important genes in CLL growth and migration such as CXCR4 (96, 97). O-GlcNAcylation of IKKβ on Ser733 from enhanced glucose metabolism prevents inactivating phosphorylation at that site and sustains TNFα-induced NFκB activation in transformed human fibroblasts (98).

In non-proliferating macrophages and hepatocytes, O-GlcNAcylation of upstream signaling inputs prevents NFκB activation (99, 100). Polymerization of RIPK3 is required for TLR4 to activate NFκB and make inflammatory molecules like TNFα or assemble necrosomes to kill *via* necroptosis. O-GlcNAcylation prevents RIPK3 from polymerizing and inhibits TLR4-signaling in murine macrophages (99, 100). CLL cells do not form necrosome complexes but it is unclear if O-GlcNAcylation of RIPK3 is responsible (101). Use of GlcN to increase O-GlcNAcylated protein levels in non-proliferative CLL cells was found to impair TLR7-signaling responses that could be enhanced by an OGT inhibitor but the relevant O-GlcNAcylated proteins were not identified (2).

### RAS, WNT, NOTCH, and MYC

The RAS/RAF/MEK/ERK, WNT/β-catenin/c-MYC, and NOTCH/c-MYC pathways are also important in CLL (16, 32, 56) and may be affected by O-GlcNAcylation. Activated RAS upregulates rate limiting enzymes of the HBP pathway, particularly GFAT2 (102, 103), and O-GlcNAcylated protein levels in cancer cells (104, 105). Mitogen-activated protein kinase kinase 2 (MEK2) can be O-GlcNAcylated at Thr13, which enhances phosphorylation at Thr394 and subsequent down-stream activation of ERK1/2 (106).

O-GlcNAcylation stabilizes β-catenin and may enhance cancer cell migration by regulating β-catenin levels (107, 108). Similarly, OGT modulates NOTCH-signaling by O-GlcNAcylating and protecting ICD from proteasomal degradation, leading to increased nuclear translocation and ICD-mediated responses (109, 110) (**Figure 2**). The WNT/β-catenin and NOTCH pathways increase transcription of *c-myc*, which mediates aggressive clinical behavior (61). O-GlcNAcylation at Thr58 is thought to stabilize MYC because phosphorylation at this site marks it for degradation. Inhibition of proteasomal destruction by O-GlcNAcylation would lead to stronger MYC activity and tumor progression (111, 112). Variable levels of O-GlcNAcylated c-MYC were seen in primary CLL cells but more samples must be studied to draw correlations with clinical course (2).

### STAT Proteins

Members of the mammalian STAT family that includes STAT1-4, STAT5a/b, and STAT6 (113) are important in CLL biology. IL10 activates STAT3 and simultaneously helps control the growth of CLL cells while contributing to the characteristic profound state of immunosuppression (114, 115). Common gamma chain binding cytokines such as IL2, IL4, and IL15 support the growth of CLL cells and are associated with STAT5A/B and STAT6 activation (116). Type 1 and type 2 IFNs activate STAT1-3 and may help control the growth of CLL cells, particularly in the presence of the bruton’s tyrosine kinase-inhibitor ibrutinib (53, 117).

Treatment of mouse mammary epithelial cells with cytokines leads to O-GlcNAcylation of STAT1, STAT3, STAT5, and STAT6 (113). The role of O-GlcNAcylation of STAT6 has not yet been explored but O-GlcNAcylation of STAT5A on Thr92 promotes activating tyrosine phosphorylation in cancer cells (118). O-GlcNAcylation stabilizes phosphorylated STAT1 to increase the duration and strength of type 2 IFN-signaling responses in mesenchymal stem cells (119). O-GlcNAcylation of STAT3 on Thr717/719 inhibits activating STAT3 tyrosine phosphorylation, preventing IL10 production by macrophages (120) and shifting neural stem cell differentiation from neurons to astrocytes (121). Inhibition of O-GlcNAcylation enhanced tyrosine-phosphorylation and STAT3 activity in these systems (120, 121).

These results suggest O-GlcNAcylation promotes STAT1 and inhibits STAT3 activity but may be context and cell-type dependent. In CLL cells, type 1 IFN strongly phosphorylates STAT1 and also phosphorylates STAT3 for various times dependent on the leukemia subtype (53). However, lowered O-GlcNAcylated protein levels were associated with inhibition of STAT3 phosphorylation that was reversed by restoring O-GlcNAcylation with glucosamine (9).

## Effects of O-GlcNAcylation on CLL Metabolism

Metabolic reprogramming is another hallmark of cancer cells (19). In contrast to resting cells that generally derive their energy from oxidative phosphorylation in mitochondria, cancer cells often exhibit the “Warburg effect” involving aerobic glycolysis that uses large amounts of glucose and glutamine for fuel and to make nucleosides, amino acids, and fatty acids for proliferation (122). Circulating CLL cells express high levels of pyruvate dehydrogenase kinase 4 (PDK4) that prevent glucose from being metabolized in the mitochondrial tricarboxylic acid cycle (37). They consequently depend on fatty acid oxidation (90) that may be linked to their immunosuppressive phenotype (123). PDK4 and fatty acid oxidation are turned off by the proliferative signals encountered in PCs (37, 90) but the increase in glycolysis is generally not high enough to be seen with 2-deoxy-2-[^18^F] fluoroglucose/positron emission tomography (FDG/PET) (124). In contrast, development of Richter’s transformation is associated with high glucose utilization detectable by FDG-PET (124).

Metabolism of cancer cells is reprogrammed by oncogenic signaling pathways and reflected by altered flux through the HBP and changes in O-GlcNAcylation (1). Aberrant O-GlcNAcylation can also directly influence the metabolic program of a cancer cell. Sustained high O-GlcNAcylated protein levels generally promote glycolysis and the Warburg effect (125) in a number of ways including O-GlcNAcylation of glycolytic enzymes. For example, the hexokinase HK1 forms glucose-6-phosphate from glucose, the initial step in most glucose-dependent metabolic pathways, and its activity is increased by O-GlcNAcylation (126).

Pyruvate kinase (PK) tetramers catalyze formation of ATP and pyruvate from ADP and phosphoenolpyruvate in the final step of glycolysis. O-GlcNAcylation of the PK isozyme PKM2 promotes less active dimerization and causes upstream glycolytic intermediates to accumulate and be routed into biosynthetic pathways such as the pentose phosphate shunt (PPP) to make ribose sugars for nucleoside formation (127). PK activity also requires binding by fructose-1,6-bisphosphate produced by phosphofructokinase 1 (PFK1). O-GlcNAcylation on Ser529 inhibits PFK1 and also directs glycolytic intermediates into the PPP (126). This O-GlcNAc modification of PFK1 may be unique to cancer cells as it was not seen in dividing normal T cells (126).

In addition to regulating glycolytic enzymes directly, O-GlcNAcylation modulates transcription factors and kinases to promote distinct metabolic programs. As described above, O-GlcNAcylation sustains the activity of c-MYC, a major inducer of glycolysis and glutaminolysis genes (128) as well as NFκB, which increases the rate of aerobic glycolysis in part by increasing transcription of glucose transporters, particularly in the absence of p53 (129).

Hypoxia-inducible factor (HIF1) regulates the expression of genes that contribute to aerobic glycolysis such as PFK1 and HK, glucose transporters such as GLUT1, and lactate dehydrogenase (LDH) (125) while carbohydrate responsive element binding protein (ChREBP) regulates PK isoforms and lipogenic enzymes such as acetyl-CoA carboxylase (ACC) (130, 131). HIF1 is composed of α and β subunits. Hydroxylation by prolyl hydroxylase domain protein 2 (PHD2) using O_2_ and α-ketoglutarate (α-KG) as substrates marks the α subunit for proteasomal degradation. Akin to hypoxia, O-GlcNAcylation prevents HIF1α destruction and upregulates HIF1 expression by inhibiting production of α-KG (125, 132). O-GlcNAcylation directly protects ChREBP from the proteasome to increase its level and transcriptional activity (130).

AKT/mTOR and AMPK are two other major regulators of the metabolic program of a cancer cell. The AKT/mTOR pathway promotes glucose uptake and utilization along with lipid and protein synthesis that are associated with the Warburg effect (122). In contrast, AMPK is activated by a low energy charge reflecting a high AMP/ATP ratio and phosphorylates substrates such as ACC1 and ACC2 to decrease lipogenesis along with TSC1/2 and Raptor to suppress mTORC1 activity and protein synthesis (133, 134).

O-GlcNAcylation of Akt has context-dependent effects and can sometimes promote or inhibit Akt activity (2, 85) but OGT and O-GlcNAcylation appear to clearly negatively regulate AMPK in solid tumor cells (125). O-GlcNAcylation of AMPK subunits inhibits kinase activity (135) and increases mTOR activity and protein synthesis (125). Inhibition of OGT by shRNA or small molecules to lower O-GlcNAcylated protein levels activated AMPK and decreased HIF1 activity along with growth and proliferation of breast cancer cells (125).

It is unclear how aberrant O-GlcNAcylation affects the metabolic program of CLL cells as they traffic from the circulation to lymph nodes and ultimately evolve a Richter’s transformation. Total O-GlcNAcylated protein levels appear to increase in lymph nodes (2) and likely increase even more as a result of increased Akt activity in a Richter’s transformation (59). Perhaps the relative decrease in O-GlcNAcylated protein levels in the circulation activates AMPK and increases fatty acid oxidation similar to the result of lowering O-GlcNAcylation in breast cancer cells (125).

## O-GlcNAcylation and T Cells

Increased T cell numbers are central to the development and evolution of CLL. CD4^+^ T cell subsets exhibit dysregulation and the ratio of CD4^+^ to CD8^+^ T cells becomes increasingly skewed with more aggressive disease (136). Th1 and Th2 cells characterized by IFN-γ and IL-4 production respectively are increased in the blood of CLL patients compared to healthy controls and Th1 cells are even higher in progressive disease (137). Numbers of Th17 cells that make IL-17 are lower than Th1 and Th2 cells but still higher than in normal controls (138) while Tregs that make IL-10 and TGF-β are increased in the blood and lymph nodes of CLL patients (139) and correlate directly with more aggressive disease (140). A decrease in Th17 cells is associated with Treg expansion and disease progression while high Th17 cell numbers correlate with improved survival (141). CD8^+^ cells are also increased in the blood of CLL patients and correlate inversely with a more benign course (142).

CD4^+^ T cells are thought to promote the growth of CLL cells. They show evidence of activation *in vivo* and IFN-γ, IL-4, and IL-17 all increase survival of CLL cells *in vitro* (117, 143, 144). CD4^+^ but not CD8^+^ T cells are required to engraft human CLL cells in immunodeficient mice (145) and cytotoxic drugs like fludarabine may work in part by depleting CD4^+^ T cells (146).

In contrast, CD8^+^ T cells are thought to protect against tumor progression. They also show evidence of *in vivo* activation and depletion of CD8^+^ T cells hastened development of CLL in a transgenic mouse model (147). However, CD8^+^ T cells become exhausted, evidenced by decreased cytokine production and cytotoxic ability along with increased expression of checkpoint molecules such as PD1, and ultimately fail to control CLL (147, 148).

The metabolic program employed by a T cell is intimately connected with its phenotype and function (149) and involves many of the factors discussed above. The AKT/mTOR pathway coordinates glycolysis, lipid synthesis, and oxidative phosphorylation in part through HIF1α and c-MYC to promote Th1 and Th2 cell differentiation (150). Similarly, AKT/mTOR positively regulates the functional differentiation of Th17 cells that exhibit aerobic glycolysis and glutamine oxidation (151). In contrast, Tregs and memory CD8^+^ T cells rely more on AMPK and fatty acid oxidation (123, 152). T cell exhaustion is accompanied by metabolic dysregulation consisting of inhibited AKT/mTOR signaling, suppressed glycolysis, limited spare respiratory capacity, and dysregulated mitochondrial function causing oxidative stress (153).

Given its relationship with cellular metabolism, aberrant O-GlcNAcylation may also be associated with T cell defects in CLL. Activation of T cells is associated with O-GlcNAcylation of over 1000 proteins (4, 154) including c-MYC and NFAT (2, 155). When O-GlcNAcylation is blocked, T cells fail to increase c-MYC, produce cytokines, proliferate, or undergo proper thymic development in mice (156). Functional cytotoxic CD8^+^ effector T cells require strong HBP activity and contain higher amounts of UDP-GlcNAc than activated CD4^+^ T cells that are both higher than naïve T cells in mice (156).

O-GlcNAcylation is also involved in the differentiation of T cell subsets. Th2 differentiation requires activation of the mTOR complex mTORC2 (157), which leads to upregulation of GFAT1 in part by regulating the expression of XBP1s (15, 158). STAT5 activity also promotes Th2 differentiation (159) and is enhanced by O-GlcNAcylation (118).

Upregulation of O-GlcNAcylated protein levels by OGA inhibition in CD4^+^ T cells promoted Th17 function evidenced by increased IL-17 production. Fatty acid and cholesterol ligands for retinoic acid receptor-related orphan receptor gamma (RORγt), the lineage-defining transcription factor of Th17 cells, were increased by enhanced activity of O-GlcNAcylated ACC1, the rate-limiting enzyme in lipogenesis (160). Upregulation of NFκB activity by O-GlcNAcylation (94, 95) may also increase transcription of RORγt (161). Down-regulation of miR-15b, which negatively regulates *OGT* gene expression, is associated with increased Th17 cells in multiple sclerosis (162).

O-GlcNAcylation is also required for lineage stability and function of Tregs. Genetic deletion of OGT in Treg cells destabilized the lineage-defining transcription factor FoxP3, inhibited IL-2-mediated STAT5 activation that regulates functional capabilities of Tregs, and produced a severe inflammatory phenotype in mice (163).

How O-GlcNAcylation selectively promotes the differentiation of myriad Th subsets is not clear. The magnitude of total O-GlcNAcylation may confer some specificity similar to the switch from glycolysis to fatty acid oxidation that occurs when O-GlcNAcylated protein levels are lowered in breast cancer cells (125) or when CLL cells exit a lymph node (90). For example, Th17 cells and Tregs are linked in that they both require TGF-β to develop from precursors (164). Addition of the pro-inflammatory cytokine IL-6 diverts T cell differentiation from the Treg pathway toward the Th17 pathway (165). Perhaps increased glucose uptake imposed by IL-6 (166) leads to higher O-GlcNAcylated protein levels and a switch to the Th17 phenotype.

Limited information is available about the functional role of O-GlcNAcylation in CD8^+^ T cells and T cell exhaustion. Down-regulation of glycolysis in exhausted T cells (153) might lead to decreased HBP flux and lower O-GlcNAcylated protein levels. Highly glycolytic tumor cells can “steal” glucose from T cells and potentially lower O-GlcNAcylated protein expression together with mTOR activity, glycolysis, and cytokine production (167) but this mechanism seems unlikely to occur in CLL in the absence of Richter’s transformation (124). CLL cells make factors including exosomes (168) that cause CD8^+^ T cells to lose glucose transporter expression and mitochondrial mass and undergo oxidative stress consistent with features of exhaustion (148). Evaluation of the public database GSE8835 (169) suggests *OGT*, *OGA* and *GFPT1* expression trend downward in healthy CD4^+^ T cells and are significantly down-regulated in CD8^+^ T cells when they are cultured with CLL cells (170).

### Tumor Associated Macrophages (TAMs)

In addition to CLL cells and T cells (**Figure 2**), O-GlcNAcylation affects other cell-types in the tumor microenvironment such as TAMs that are an important source of oncogenic and immunosuppressive factors. Inflammatory cytokine production by TLR-activated macrophages is limited by O-GlcNAcylation (99, 171). TAMs have properties of M2 macrophages (45, 46) and OGT mediates M2 polarization in human macrophages (172). Increased UDP-GlcNAc has been associated with M2 polarization of murine macrophages (173) but another study found OGT and O-GlcNAcylation did not affect the M2 pathway although M1 polarization was suppressed by inhibiting S6K1 through O-GlcNAcylation of Ser489 (174).

## O-GlcNAcylation in Other Hematological Malignancies

Aberrant O-GlcNAcylation appears to be a feature of other hematological malignancies, including myelodysplastic syndromes (MDS), acute myeloid leukemia (AML), acute lymphoblastic leukemia (ALL), mantle cell lymphoma (MCL), diffuse large cell lymphoma (DLCL), and multiple myeloma (MM). A lack of data, some of which appears contradictory, currently limits the understanding of the role of O-GlcNAcylation in blood cancers. Interestingly, cell-lines from hematological malignancies express higher levels of *OGT* compared to all other solid tumor cell lines (22). Moreover, high *OGT* expression in primary cells is associated with an adverse clinical outcome in DLCL and MM as well as CLL (**Figure 3A**), although a complete picture of the role of O-GlcNAcylation requires information on OGT and OGA protein expression along with a catalogue of the cancer specific O-GlcNAcome. While this association suggests OGT is a tumor promoter and novel therapeutic target, there is also evidence in AML and MDS that O-GlcNAcylation helps slow disease progression.

### AML

Primary AML cells have increased OGT and GFAT1 expression compared to normal PBMCs (175). Inhibition of HBP activity with the purported GFAT inhibitor DON (13) resulted in growth arrest, differentiation, and death of OCI-AML3 and HL60 AML cell lines *in vitro* and decreased growth of HL-60 cells in immunodeficient mice without major toxicity (175). Resistance to chemotherapy was also associated with increased O-GlcNAcylated protein levels and the OGT-inhibitor OSMI-1 enhanced killing by doxorubicin in HL-60 cells and primary AML cells from patients with recurrent or resistant disease (176). Similarly, resveratrol inhibited growth of erythroleukemia cells in immunocompetent mice associated with down-regulation of O-GlcNAcylated proteins (9). Taken together, these results suggest OGT and O-GlcNAcylation may promote the growth of AML cells and constitute therapeutic targets in this cancer.

In contrast, OGT was found to stabilize ASXL1 by O-GlcNAcylation on Ser199 (177). ASXL1 is a tumor suppressor in hematologic malignancies that activates gene expression by methylating histones and forming the H3K4me3 mark associated with active transcription. Impaired activity of mutated ASXL1 is associated with failure of differentiation and development of MDS and AML. Knockdown of OGT prevented differentiation of HL-60 cells in response to ATRA while the OGA inhibitor PUGNAC increased O-GlcNAcylation and promoted differentiation as indicated by expression of the myeloid marker CD11b. Pretreatment with PUGNAC also prevented engraftment of leukemic cell-lines expressing a mutant form of ASXL1 in immunodeficient mice but not leukemic cells expressing another oncogene (177). Consistent with these findings, differentiation of leukemic blasts by cannabinoids produced clinical responses associated with increased expression of OGT *in vitro* and *in vivo* (178). Cannabinoid-mediated differentiation of Jurkat and MOLMM14 cell-lines used to model acute leukemia *in vitro* was blocked by gene-silencing of *OGT* and enhanced by increasing O-GlcNAcylation with the OGA inhibitor TMG (178).

The findings that inhibition of O-GlcNAcylation can both induce (175) and prevent (177, 178) differentiation of AML cells suggest OGT- and O-GlcNAc moieties may promote MDS and AML in some conditions and suppress it in others. Reasons for these discordant results are unclear. It may be that O-GlcNAcylation changes microenvironmental conditions induced by different chemotherapeutic drugs or has different outcomes depending on the stage of disease. For example, OGT and O-GlcNAcylation may have tumor suppressor activity early in the course of MDS and AML when ASXL1 is intact (179) but acquire tumor promoting activity when *ASXL1* is inactivated by mutation. O-GlcNAcylation may also have different effects on specific AML subtypes and a detailed characterization of the genetic makeup of the AML cell or perhaps consideration of the state of the p53 axis as in CLL (62) may be required to know if inhibition or enhancement of O-GlcNAcylation is the appropriate treatment strategy.

### ALL

Less information is available about ALL. OGT and O-GlcNAcylated proteins are increased while OGA is decreased in CD19^+^ cells from B-ALL patients compared to healthy donors (85). O-GlcNAcylated protein levels correlated directly with LDH in blood, perhaps reflecting enhanced glycolysis in more aggressive leukemia cells due to high levels of PI3K activity, phospho-AKT, and c-MYC (85). Inhibition of OGT lowered glycolysis and the PI3K/AKT/MYC axis in NALM-6 cells used to model ALL (85).

### Lymphoma

OGT transcripts and protein levels are higher in DLCL cell lines and *OGT* mRNA is higher in primary DLCL cells than normal B cells (127, 180). Nuclear O-GlcNAcylated proteins are also higher in DLCL cell lines and primary cells (180). High *OGT* expression is associated with poor responses to chemotherapy (180) (**Figure 3**) and expression of *OGA* was also inversely associated with survival of DLCL patients (181). OGT was shown to O-GlcNAcylate pyruvate kinase M2 (PKM2), forcing formation of the dimer that shunts glucose into the pentose phosphate pathway to increase proliferation and growth of cancer cells (127). Inhibition of HBP pathway and O-GlcNAcylation with azaserine, another purported competitive GFAT inhibitor, down-regulated NFκB activity and increased killing of DLCL cell-lines. Cells with higher expression of OGT were more sensitive to azaserine (180).

As with AML, other forms of lymphoma gave opposite results. MCL cell-lines and primary cells were protected from bortezomib by alloxan used as an OGT inhibitor. Alloxan is not specific for OGT but the authors also showed the MCL cells were sensitized by several OGA inhibitors including PUGNAC, TMG, and ketoconazole. O-GlcNAcylation prevented degradation of tBID and increased its interactions with BCL2 family members and BAK to cause mitochondrial permeabilization and apoptosis (181).

### MM

O-GlcNAcylated proteins may be especially important in myeloma given the role of XBP1 in both the pathogenesis of this blood cancer (182) and the hexosamine pathway (15). OGT levels are high in hematological cancer cell-lines and highest in myeloma models (22). Moreover, analysis of the GEO dataset GSE24080 suggests high *OGT* mRNA expression in primary myeloma cells is associated with an adverse outcome (183).

Public databases suggest O-GlcNAcylation is actually reduced in myeloma as *OGA* was significantly higher in 133 primary myeloma samples compared to normal plasma cells from 5 normal donors with similar *OGT* expression (184, 185). Myeloma microenvironments contain high levels of calcium released by osteolysis. Calcium-signaling in myeloma cell lines was found to decrease total O-GlcNAcylated protein levels associated with up-regulation of the integrins ITGB7 and ITGA4, enhanced motility, and more aggressive clinical behavior. Increasing O-GlcNAcylation by inhibiting calcium-signaling or genetic ablation of *OGA* resulted in proteasomal degradation of the integrins, decreased motility and decreased growth of RPMI8226 myeloma cells in immunodeficient mice (184). These findings recall results in CLL where higher O-GlcNAcylated protein levels are associated with a more indolent clinical course (2) and suggest disease progression might be slowed by strategies to increase O-GlcNAcylated protein levels in MM cells. In contrast, resistance of RPMI8226 cells to bortezomib, a drug used commonly to treat myeloma, was associated with increased O-GlcNAcylated protein levels and reversed by inhibiting OGT (186).

Overall, O-GlcNAcylation appears to have different effects in cancer cells depending on underlying genetic events and metabolic environments. Therapeutic manipulation of O-GlcNAcylation holds promise in blood cancers but requires knowing when O-GlcNAcylation has tumor promoting or tumor suppressing effects.

## O-GlcNAcylation as a Tumor Promoter

Dysregulation of signaling pathways by O-GlcNAcylation orchestrates programs that promote growth and survival of cancer cells. Enhanced AKT-signaling by O-GlcNAcylation (85) inhibits apoptosis by phosphorylating the pro-apoptotic protein Bad and preventing caspase-3 activation (187). Enhanced NFκB activity (92, 94, 96, 188) prevents apoptosis by upregulating MCL-1 (189). O-GlcNAcylation can inhibit cleavage of apoptotic caspases, necroptosis (99, 100, 190), and TRAIL-mediated death by preventing oligomerization of DR5 and transmission of death-receptor signaling (191). Promotion of cancer cell proliferation and inhibition of apoptosis by O-GlcNAcylation is further supported by OGT-mediated O-GlcNAcylation and stabilization of the polycomb group transcription repressor Bmi-1, which inhibits transcription of p53, PTEN, and CDKN1A/CDKN2A genes and prevents their tumor suppressive activities (192).

The link between endoplasmic reticulum (ER) stress and the HBP (14, 15) suggests O-GlcNAcylation may enable cancer cells to survive in harsh microenvironmental environments. Limited nutrients and cytotoxic agents in the microenvironment impose stresses on cancer cells (25). O-GlcNAcylation affects metabolic changes that allow cancer cells to thrive in harsh conditions. For example, O-GlcNAcylation at Ser172 of the glycolytic regulator 6-phosphofructo-2-kinase/fructose-2,6-bisphosphatase (PFKFB3) is needed for tumor proliferation in hypoxic conditions. This modification promotes nuclear localization of PKFB3, which then prevents build-up of hypoxia-induced-P27 to allow growth in these conditions (193). O-GlcNAcylation of glucose-6-phosphate dehydrogenase (G6PD) promotes the pentose phosphate pathway that produces antioxidants and ribose sugars for nucletotide synthesis to support tumor growth in hypoxia (194). O-GlcNAcylation of fumarase (FH) prevents FH-catalyzed fumarate from inhibiting KDM2A demethylase activity that would otherwise facilitate expression of genes that mediate cell-cycle arrest in low-glucose (195).

Upregulation of O-GlcNAcylated proteins may also overcome oxidative and ER stresses imposed in the cancer microenvironment by radiation and cytotoxic drugs. Oxidative stress causes DNA damage that can be repaired by the HBP and OGT through O-GlcNAcylation of H2AX, H2AS40, and H2B histones, the Polycomb Related Complex 2 (PRK2) HMT catalytic subunit Ezh2, and the scaffold protein MDC1 to prevent radiation-induced senescence and cell-death (196, 197). Blocking OGT increases sensitivity to oxidative stress and DNA damage, leading to apoptosis or senescence and preventing tumor progression (196, 198).

ER stress in CLL cells is partly related to signaling through the BCR (199, 200) and may increase HBP activity through a IRE1/XBP1s/GFAT axis that can protect cells from death (15). Inhibition of this O-GlcNAcylation-mediated regulatory loop helps sensitize cancer cells to stress (201). Taken together, these observations are consistent with a view that O-GlcNAcylation may sustain cancer cells and that blocking OGT and the hexosamine pathway offers a novel approach to treatment for many cancers including CLL.

## O-GlcNAcylation as a Tumor Suppressor

Situations also exist in which O-GlcNAcylation has anti-tumor activity. As described above, O-GlcNAcylation potentiates the tumor suppressive activity of wild-type p53 (80, 81) and down-regulates AKT and NFκB activity in non-proliferating CLL cells (2) (**Figure 2**). Stabilization of MYC by O-GlcNAcylation promotes cell growth under nutrient-rich conditions but causes activation-induced death in nutrient- and growth factor-poor conditions that can exist in a tumor microenvironment (202). Low OGT activity promotes c-MYC degradation to maintain survival in low glucose of cancer cells that were killed when O-GlcNAcylation was increased by the OGA inhibitor PUGNAC (203). Blocking OGT would then be expected to protect cancer cells in these conditions.

Nuclear factor erythroid 2 like 2 (NRF2) encoded by *NFE2L2* is a master regulator of antioxidants that protect cancer cells from oxidative stress (25). KEAP1 is the primary negative regulator of NRF2 and OGT-mediated O-GlcNAcylation at Ser104 is required for efficient ubiquitination and degradation of NRF2. Glucose deprivation lowers O-GlcNAcylation and stabilizes NRF2, allowing cancer cells to survive in harsh conditions. The effects of low glucose on both O-GlcNAcylated proteins and NRF2 levels can be overcome by adding GlcNAc or GlcN to increase UDP-GlcNAc or by inhibiting OGA (204).

Autophagy is another mechanism employed by cancer cells to survive in nutritionally poor conditions. Autophagy is promoted by the formation of a SNARE complex containing the protein SNAP-29 that fuses autophagosomes with endosomes and lysomes. O-GlcNAcylation of SNAP-29 prevents assembly of the complex and negatively regulates autophagy (205, 206) in solid tumors. It is unclear if this mechanism applies to CLL cells or other hematological cancers.

## O-GlcNAcylation as a Therapeutic Target

### Blocking O-GlcNAcylation

Strategies to block OGT activity include direct inhibitors of OGT (10) and HBP enzymes, particularly GFAT (13). XBP1 inhibitors may also block O-GlcNAcylation (207) given XBP1 is a transcriptional regulator of GFAT (15). Flavonoid compounds like resveratrol can induce rapid proteasomal clearance of O-GlcNAcylated proteins from CLL cells (9).

Specific targeting of glycosyltransferases is difficult and no OGT inhibitors are currently available for clinical use. However, new inhibitors like OSMI-4 have low nanomolar potency and less off-target activity than previous ones (2, 10), suggesting they may form the basis for future phase 1 testing. 5SGlcNAc (11, 12) potently decreases O-GlcNAcylated proteins and UDP-GlcNAc *in vitro* by generating UDP-5SGlcNAc, a competitive OGT inhibitor with Ki=8 μM (11, 12). Poor aqueous solubility limits the use of such metabolic inhibitors but new analogs have greater activity *in vivo* and may ultimately be incorporated into leukemia treatment strategies (12). The glutamine antagonist DON inhibits GFAT non-specifically and has anti-cancer activity *in vivo* at the expense of toxicity that may be minimized with lower concentrations in novel dosing strategies (13).

Inhibition of OGT and O-GlcNAcylation prevent progression of a number of solid tumors (22, 102). Genetic ablation of OGT in pre-B cells downregulates c-MYC and prevents B cell development in mice (91) suggesting OGT may be a novel therapeutic target for B cell cancers such as ALL. Observations that resveratrol decreased O-GlcNAcylated protein levels and slowed tumor growth in CLL patients provide more support for O-GlcNAcylation inhibitors in leukemia (9). However, the anti-leukemic effects were transient and overcome by factors such as IFN that stimulated HBP activity (9). 5SGlcNAc analogues also had only transient effects *in vivo* (11, 12). Direct inhibition of OGT causes increased transcription of a reservoir pool of non-spliced *OGT* mRNA to restore O-GlcNAcylation (10). Accordingly, OGT inhibitors may be most effective when used with other therapeutic strategies including combination with GFAT inhibitors.

Consistent with this idea, inhibition of OGT enhances killing of solid tumor cells by conventional chemotherapeutic agents such as anthracyclines (176, 208). OGT promotes NRF1-mediated up-regulation of proteasome subunits (209), which may account in part for the intrinsic resistance of CLL cells to the proteasome inhibitor bortezomib and suggesting it may be overcome by inhibiting OGT (210). OGT inhibitors improve killing of cancer cells by PI3K inhibitors, suggesting OGT mediates resistance to PI3K inhibitors and possibly identifying a strategy to improve outcomes with kinase inhibitors in CLL patients (211, 212).

### Enhancing O-GlcNAcylation

In contrast to OGT, OGA inhibitors are available for clinical use. MK-8719 has orphan drug status in the USA for the neurological condition progressive supranuclear palsy (PSP) but has not yet been explored in leukemia (213).

Cancer cells may be sensitized to chemotherapy in some situations by increasing O-GlcNAcylated proteins and protected by inhibiting O-GlcNAcylation. The status of the p53 axis may perhaps indicate these situations as wildtype p53 activity is potentiated by O-GlcNAcylation (80, 81). The OGA inhibitor TMG sensitizes human leukemia cells to the microtubule inhibitor paclitaxel (214) and mantle cell lymphoma cells to bortezomib (181). HBP- and OGT-inhibitors promote resistance of ovarian cancer cells to platinum-based chemotherapy by inducing autophagy (205, 206) and secretion of exosomes that decrease intracellular drug levels (215). O-GlcNAcylation of the death receptor DR4 on Ser424 sensitizes cancer cells to TRAIL-mediated apoptosis and necrosis. TRAIL-induced apoptosis is then increased by the OGA inhibitor TMG but prevented by the OGT inhibitor ST04589 (216).

Glucocorticoids (GCs) remain important drugs for blood cancers including CLL (217) and OGT has been shown to bind the glucocorticoid receptor (GR) and mediate transrepression of NFκB by O-GlcNAcylating RNA polymerase II (218). Inhibition of OGT prevents GC-mediated apoptosis (218).

Taken together, these observations suggest more detailed knowledge of its distinct role in the biological processes that underlie cancer hallmarks is still required to effectively manipulate O-GlcNAcylation as a treatment strategy (19). Global inhibition or enhancement of O-GlcNAcylation may not be the best treatment strategy as OGT is the only enzyme in humans capable of transferring GlcNAc moieties to proteins and inhibiting OGT may cause major toxicities. Consistent with this, mice injected with high doses of 5SGlcNHex became moribund although lower amounts were better tolerated (12). Given the role of O-GlcNAcylation in T cell biology (4, 156, 160), direct OGT inhibition may also cause significant immunosuppression. O-GlcNAcylated and de-glycosylated proteins can both mediate survival of cancer cells in different conditions and cancer cells with intact p53 functioning that may respond to increasing O-GlcNAcylation (81) could possibly be accelerated by HBP and OGT inhibition. If OGT-activity promotes the development and progression of cancers with impaired p53 axes but decreased OGT-activity helps them live in harsh microenvironments, a useful strategy in such cases might involve brief treatments with OGT inhibitors followed by infusion of GlcN or OGA inhibitors to block survival processes induced by prior OGT-inhibition. Another option might be to try to identify cancer cell specific vulnerabilities that are lethal in the presence of a low dose of an OGT inhibitor. For example, the GFAT2 inhibitor cycloserine was found to exhibit synthetic lethality with an OGT inhibitor in prostate cancer cells (219).

## Summary and Future Work

Tremendous progress in the understanding of O-GlcNAcylation and its role in cancer has been made in the last decade. O-GlcNAcylation has emerged as a new cancer “hallmark” with an established role in a number of central oncogenic processes. Targeting O-GlcNAcylation offers a novel approach to improve results of current therapies and the lives of patients with hematological and other cancers.

Despite this progress, much remains to be done. More information is needed about how O-GlcNAcylation affects different blood cancers. It seems clear that O-GlcNAcylation can suppress or promote cancer development depending on the stage of disease, type of cancer, and microenvironmental conditions. Development of better mouse models might help clarify the effect of O-GlcNAc during oncogenesis. For example, the role of OGT as a tumor promoter or suppressor in CLL could be addressed in more detail by B cell lineage specific deletion of OGT in TCL1-transgenic mice, considered an excellent model of aggressive *IGHV* unmutated CLL (59, 200).

Cellular processes affected by aberrant O-GlcNAcylation in different cancers require additional investigation. OGT and O-GlcNAcylation are regulated by cell type-specific mechanisms so that results in one cancer do not necessarily generalize to others. Simply cataloguing proteins that are O-GlcNAcylated in different cancers and in different microenvironments would be helpful to implicate affected processes. Advances in chemoenzymatic labeling strategies for identifying O-GlcNAc modifications by mass-spectrometry (7, 190) make this possible and the results should be correlated with underlying genetic aberrations of the cancer cells.

Also needed is more detailed understanding of how O-GlcNAcylation affects important oncogenic signaling processes. Precise genetic subtyping of individual leukemia cells is required as different types have different levels of O-GlcNAcylation and protein targets that may have disparate effects on signaling responses. These studies should also be done in conditions that more accurately reflect conditions in a cancer microenvironment, including hypoxia, low-glucose, and three-dimensions (220). The role of O-GlcNAcylation in the T cell compartment of CLL (**Figure 2**) and its relationship to failure to clear leukemia cells also requires further study.

Clinically relevant OGT inhibitors may soon be available (10). As discussed above, results of globally increasing or decreasing O-GlcNAc levels may be limited but short-term blockade of OGT to minimize toxicity may synergize with many cytotoxic drugs and kinase inhibitors. Appropriate modulation of O-GlcNAcylation in T cells may lead to improved immunotherapies (221). The apparent importance of O-GlcNAcylation in hematologic malignancies suggests they may be useful models for evaluating OGT modulators in clinical trials.

## Author Contributions 

The author confirms being the sole contributor of this work and has approved it for publication.

## Funding

This work was supported by CIHR grant #374817 and the Leukemia and Lymphoma Society of Canada (LLSC).

## Conflict of Interest

The author declares that the research was conducted in the absence of any commercial or financial relationships that could be construed as a potential conflict of interest.

## Publisher’s Note

All claims expressed in this article are solely those of the authors and do not necessarily represent those of their affiliated organizations, or those of the publisher, the editors and the reviewers. Any product that may be evaluated in this article, or claim that may be made by its manufacturer, is not guaranteed or endorsed by the publisher.
